# Longitudinal retinal imaging study of newly diagnosed relapsing-remitting multiple sclerosis in Scottish population: baseline and 12 months follow-up profile of FutureMS retinal imaging cohort

**DOI:** 10.1136/bmjophth-2022-001024

**Published:** 2022-07-22

**Authors:** Yingdi Chen, Juan Larraz, Michael Wong, Patrick Kearns, Fraser Brown, Sarah-Jane Martin, Peter Connick, Niall MacDougall, Christine Weaver, Baljean Dhillon, Siddharthan Chandran

**Affiliations:** 1 The Anne Rowling Regenerative Neurology Clinic, The University of Edinburgh, EdinburghUK; 2 Centre for Clinical Brain Sciences, University of Edinburgh, Edinburgh, UK; 3 MRC Human Genetics Unit, Chromatin Lab, Genome Regulation Section, University of Edinburgh, Edinburgh, UK; 4 Department of Neurology, Institute of Neurological Sciences, Queen Elizabeth University Hospital-, University of Glasgow, Glasgow, UK; 5 Department of Clinical Neurosciences, Edinburgh Royal Infirmary, NHS Lothian, EdinburghUK; 6 Department of Neurology, University Hospital Hairmyres, East Kilbride, UK; 7 Princess Alexandra Eye Pavilion, NHS Lothian, EdinburghUK; 8 UK Dementia Research Institue, The University of Edinburgh, Edinburgh, UK

**Keywords:** Imaging, Diagnostic tests/Investigation, Degeneration

## Abstract

**Objective:**

Multiple sclerosis (MS) is an inflammatory degenerative condition of central nervous system. The disease course and presentation of MS is highly heterogeneous. Advanced retinal imaging techniques such as optic coherence tomography (OCT) can capture abnormalities of anterior visual pathway with high resolution, which may contribute greater insights into the pathophysiology of MS.

**Methods:**

People with newly diagnosed relapsing-remitting MS were recruited for FutureMS retinal imaging study from two study centres in Scotland. The baseline visit was completed within 6 months of diagnosis with initial follow-up 12 months after the baseline visit. The assessments included in FutureMS retinal imaging study were visual acuity test, self-reported eye questionnaire and OCT scan.

**Results:**

A total of 196 FutureMS participants completed the retinal imaging study of FutureMS with 185 participants at M0 and 155 at M12. A total of 144 participants completed both M0 and M12 visits. At the whole cohort level, the distribution of retinal measures is generally consistent between baseline and follow-up.

**Conclusion:**

The FutureMS retinal imaging study aims to demonstrate that patient with MS present with different extent of retinal abnormalities that can be captured by retinal imaging modalities such as OCT soon after diagnosis. These changes may sensitively mirror the brain atrophy or serve as predictors for disease activity. By developing sensitive, quantifiable and objective retinal biomarkers, FutureMS retinal imaging study will provide an opportunity to stratify patient with MS at an early stage and support future therapeutic strategies for a better outcome.

**What is already known on this topic**

- Optic coherence tomography (OCT) images have potential value in diagnosing multiple sclerosis (MS).

**What this study adds**

- This study aims to establish a rich dataset for evaluating in vivo visual pathway changes.

**How this study might affect research, practice or policy**

- This study provides opportunities for deep phenotyping of patients with MS via combining OCT parameters with other clinical and imaging biomarkers.

## Introduction

Multiple sclerosis (MS) is a chronic inflammatory and degenerative disorder of the central nervous system (CNS) that affects c.2.5M globally. It is the leading non-traumatic cause of neurological disability among young adults in Europe and the USA, but exhibits substantial variability between individuals in its features and course. Relapsing-remitting MS (RRMS) is the most common form affecting about 85% of patients with MS. It is characterised by discrete episodes of exacerbations followed by symptom free period. The remaining 10%–15% of individuals present with progressive course from the onset. Despite advances in brain imaging and clinical biomarkers, no scalable approach currently exists that can accurately predict disease evolution for individual people with MS; this is a major unmet need for the application of precision medicine in MS.

FutureMS (https://future-ms.org/) is a large, national-representative longitudinal observational cohort study with newly diagnosed relapsing remitting MS recruited in Scotland.^1^ The clinical^1^ and radiological^2^ protocols for the study have been described in details elsewhere. This study is designed in waves with participants recruited for baseline recruitment within 6 months of diagnosis and prior to starting any disease-modifying therapies. Initial follow-up has been completed at 12 months postbaseline for all participants. Further follow-up is planned at 5 years after baseline as part of an extension study. Participants undergo assessments including clinical, laboratory and imaging at each visit with broader assessments including genetic parameters at baseline. Such a cohort gives us an opportunity to understand the long-term evolution of MS. The aim of this study is to develop predictive tools for personalised care for people with MS.

The anterior visual pathway consists of retinal ganglion cells and their axons that form the optic nerve and travel to the lateral geniculate nucleus of the thalamus. The anterior visual pathway is highly susceptible to neuroinflammatory episodes in MS. Visual symptoms are common at the time of diagnosis in MS and about 25% of people with RRMS have optic neuritis (ON) as their initial symptom.^3^ Although many patient with MS never clinically manifest episode of ON, postmortem studies confirm that optic nerve involvement is present in almost 99% of cases.^4^ Optic coherence tomography (OCT) is a fast, low cost, non-invasive imaging technique that can capture the cross-sectional tomographical imaging of the retina and optic nerve with high resolution and high reproducibility. With its easy accessibility by modern imaging technologies, anterior visual pathway provides a readily observable component of the CNS.

The retina has an elaborate laminated structure with each layer playing a specific role in the visual pathway. The abnormalities in different retinal layer may reflect certain aspect of pathophysiological process. Retinal nerve fibre layer is the innermost of the retina and is formed with axons of retinal ganglion cells. The retinal nerve fibres within the eye are non-myelinated, therefore, it is a perfect site to evaluate neuron axonal damage. Evidence has shown that the anterior visual pathway follows a bidirectional transsynaptic axonal degeneration, therefore, changes in retinal nerve fibre may reflect degeneration process in the visual pathway.^5^ The retinal ganglion cells are third-order neurons reside in the ganglion cell layer (GCL). Retinal ganglion cells are known to be vulnerable in neurological conditions including MS, and the GCL is considered to be an ideal site to investigate neurodegeneration because of their easy accessibility via OCT imaging. Due to the low contrast difference, GCL is often quantified together with inner plexiform layer (dendrites of RGCs and synapses of bipolar cells) as the ganglion cell inner plexiform complex (GCIPL). With the highest density of retinal ganglion cells, the GCIPL constitutes about one-third of the macular volume, is considered to be a promising biomarker for neurodegeneration. Evidence has shown that GCIPL thinning is associated with intrathecal B-cell immunity (B cell number and immunoglobulin index) and worsening disability in MS.^6^

The inner nuclear layer consists of cell bodies of bipolar cells, which are the second order neurons that connect inner and outer retina. The value of inner nuclear layer as a biomarker for MS has been recognised in recent years. Abnormalities in INL that often presented in the form of either microcystic macular oedema or tissue thickening, are reported to be associated with recent MS disease activity and neuroinflammatory disease activity on MR imaging.^6–8^ Hence, INL thickness may be a useful tool to predict neuroinflammatory disease activity and progression. The outer nuclear layer (ONL) contains the cell bodies of the first order neurons, the photoreceptors. It used to believe that the ONL is unaffected in MS except a temporal swelling on ON attack.^9^ Recent evidence suggests that, despite normal appearance in OCT imaging, abnormalities in the outer retina are frequently indicated by functional testing.^10^ This may be because OCT imaging, similar to MRI imaging, does not discriminate dysfunctional and healthy cells in the tissue. Therefore, it added valuable may arise from combining OCT imaging with functional testing when assessing the abnormalities in the outer retina.

FutureMS incorporates a standard OCT protocol that provides qualitative and quantitative assessment of the structure and vasculature in the retina and optic nerve head. The aim of this paper is to provide a rationale and transparent overview of OCT acquisition and processing in this study and introduce the profile of the participants in the OCT imaging study.

## Cohort description

### Study population and recruitment

FutureMS is a national prospective observation cohort study of individuals with newly diagnosed RRMS. A substudy for retinal imaging including OCT scan, 100% and 2.5% visual acuity test as well as a self-reported eye questionnaire was conducted in two assessment centre where devices were available. The FutureMS retinal imaging study was offered to FutureMS participants in Edinburgh site who entered the study after April 2017 and participants in Glasgow site who entered the study after December 2017. Eligibility criteria were as for the main FutureMS study: Diagnosis confirmed by the treating consultant neurologist as fulfilling the most recent McDonald Criteria for RRMS in the previous 6 months and no usage of a disease-modifying therapy prior to baseline assessment, capacity to give informed consent and no contraindication to MR brain imaging at the time of their baseline visit.

At study entry (M0), participants had ophthalmic assessment at the same day as the data collection for the rest of the study including blood test and MRI scan, and then repeat at 12 months (M12). The recruitment for baseline visit was carried out between April 2017 and October 2019 in Edinburgh and between December 2017 and August 2019 in Glasgow. The 1-year follow-up visit was carried out between August 2018 and March 2020 in Edinburgh and between December 2018 and February 2020 in Glasgow.

### OCT acquisition protocol

OCT images were acquired at two sites following Advised Protocol for Optical Coherence Tomography Study Terminology and Elements recommendations.^11^ Scan on both sites were performed using a spectral domain OCT device (Spectralis, Heidelberg Engineering). In August 2018, OCT device in Edinburgh site was upgraded from the first generation of the Spectralis SD-OCT device (OCT1) to the second generation device (OCT2). On upgrading, all FutureMS study data collected at this point were exported from OCT1 device in Heidelberg raw file format (.E2E) and imported into OCT2 device to enable the follow-up function at M12. The scanning protocol remained the same after upgrade. Detailed information on acquisition device are shown in online supplemental table 1.

10.1136/bmjophth-2022-001024.supp1 Supplementary data

All participants underwent a standard OCT protocol on both eyes. OCT scans were performed in a room with consistent illumination by trained operators on both eyes of the participants without pupil dilation. For each eye, the scanning protocols include a horizontal single line scan, a vertical single line scan, a posterior pole volume scan for macular area and a circle scan for peripapillary area. Detailed information in scanning protocols are shown in online supplemental table 2.

### Quality control and data cleaning

All data were anonymised before being transferred to the retinal imaging research team at Edinburgh. All records have gone through two steps of assessment. The initial step was the pathological review done by an experienced ophthalmologist (YC and BD). The pathological assessment was completed based on self-reported eye questionnaire and OCT images. Records with substantial retinal pathologies as defined in OSCAR-IB criteria were excluded.^12^ All records passed pathology assessment then went through thorough imaging quality assessment by experienced raters (YC, JL and MW). Each image was assessed by a single rater, with a subset repeated by a second rater to ensure reproducibility.

Images passed both steps of assessment were included in following data processing steps. Segmentation for protocol 3 and protocol 4 were automated using Eye Explorer software (Heidelberg Spectralis, V.6.0.9.0), and then checked manually and corrected where necessary in a blinded manner by a trained examiner (YC, JL and MW). The measurements were calculated using software algorithm.

### Statistics

Statistical analysis will include descriptive analysis to compare the mean, median, frequencies, percentages and SD. Generalised mixed effect regression will be used to investigate the relationship between variables and will be describe elsewhere. For this study, data analysis and statistics were completed using Matlab (V.R2021a, Mathworks) with the Statistics and Machine Learning toolbox. Results were presented as mean±SD, median with IQR, frequency or percentage. Distribution of retinal measures were obtained using kernel smoothing function. The comparison on retinal measures between M0 and M12 were done using one-way analysis of variance (ANOVA) with one-sample Kolmogorov-Smirnov test.

### The retinal microstructure visualised using OCT in the FutureMS cohort

In the clinical study, the assessment of retinal layers is usually completed by two standard scanning protocols, a volume scan on the posterior pole that encompasses 30°×25° of retina (FutuerMS OCT imaging Protocol 3) (online supplemental figure 1), and a 12° diameter circle scan centred on the optic disc (FutuerMS OCT imaging Protocol 4)(online supplemental figure 2). The protocol 3 outputs retinal measures from macular area based on the Early Treatment Diabetic Retinopathy Study (ETDRS) grid.^13^ Centred on fovea, the ETDRS grid delineate macula area into nine sectors that covers the area within 6 mm of fovea. The macular parameters extracted for FutureMS dataset are macular nerve fibre layer, macular GCL, macular inner plexiform layer, macular inner nuclear layer, macular ONL as well as the whole retinal layer. Each layer includes thickness measurements from the nine sectors and a perifoveal volume of corresponding layer (online supplemental figure 1). The protocol 4 outputs the thickness of circumpapillary RNFL (pRNFL). The pRNFL covers full contents of retinal axons, follows a ‘double-hump’ pattern with two peaks at superior and inferior and two troughs at temporal and nasal. The variables for pRNFL extracted for FutureMS dataset include a global average thickness, average thickness of six sectors, thickness of papillomacular bundle (PMB) and nasal/temporal ratio (online supplemental figure 2). The thickness of pRNFL was classified based on the software integrated reference database (European descent 2009, Spectralis, Heidelberg Engineering). A percentile value of <1% was labelled as below normal limit (BNL) and a percentile value of <5% was labelled as borderline below (BLB).

## Results

### Demographic and clinical characteristics

**Figure 1:**
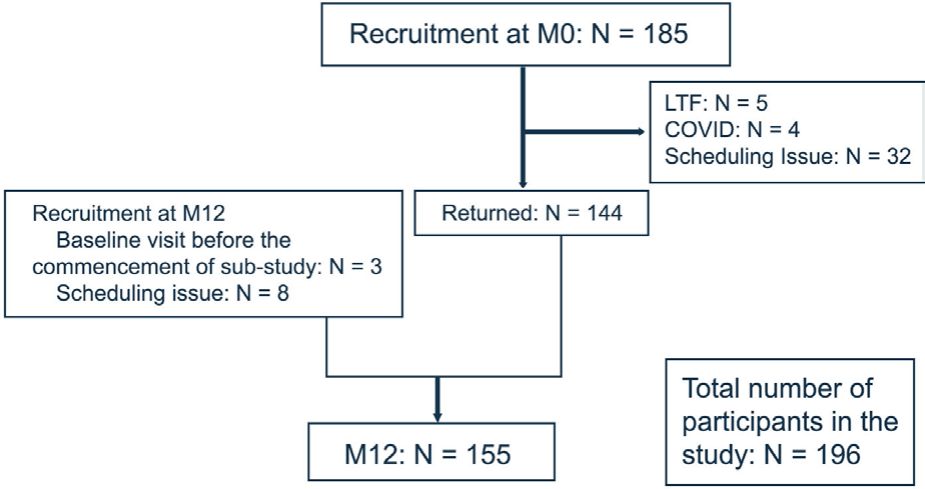
Study recruitment flow chart.Flow diagram showing the study recruitment process. LTF, loss to follow-up.

The detailed recruitment process is depicted in figure 1. A total of 440 participants were recruited in FutureMS main study, of whom 196 have consented to the substudy with 185 participants at M0 and 155 at M12. A total of 144 participants completed both M0 and M12 visits. Of the 11 participants who only had their scans at M12, 8 were due to scheduling issue and three had their baseline visit before the commencement of the substudy therefore they were enrolled at M12 visit. Forty-one participants who participated in M0 did not return for the follow-up visit at M12. Five were lost to follow-up. Four were not able to complete the study due to COVID-19. Thirty-two who completed main study assessment did not complete the substudy assessment due to scheduling issues.

The demographic and clinical characteristics for ophthalmic participants are presented in table 1.

**Table 1:** Demographic and clinical characteristic for study participants

	M0		M12	
	Edinburgh (n=98)	Glasgow (n=87)	Edinburgh (n=92)	Glasgow (n=63)
Age (years, mean±SD)	37.17±11.05	37.02±9.58	39.27±11.12	37.29±10.11
Gender (F:M)	75:23	59:28	67:25	43:20
Disease duration (days, mean±SD)	62.15±36.88	59.85±36.28		
Visual symptom at onset (n / %)			N/A	N/A
Vision loss	25/58.14%	14/53.85%		
Double vision	14/32.56%	10/38.46%		
Both	4/9.30%	2/7.69%		
EDSS score (median/IQR)	2/1.5	2/1	2.5/1.5	2.5/1
MSFC (median/IQR)	0.200/0.689	0.167/0.893	0.384/0.881	0.359/0.741
ON history (n / %)				
No history of ON	54/60.67%*	39/68.42%†	47/58.75%‡	27/72.97%§
Unilateral ON	32/35.56%	13/22.81%	28/35%	8/21.62%
Bilateral ON	3/3.37%	3/8.77%	5/6.25%	3/5.41%

*Edinburgh site (M0): Total number of 89 participants have ON history available based on self-reported eye questionnaire and clinical records.

†Glasgow site (M0): Total number of 55 participants have ON history available based on self-reported eye questionnaire and clinical records.

‡Edinburgh site (M12): Total number of 80 participants have ON history available based on self-reported eye questionnaire and clinical records.

§Glasgow site (M12): Total number of 38 participants have ON history available based on self-reported eye questionnaire and clinical records.

EDSS, Expanding Disability Status Scale; MSFC, Multiple Sclerosis Functional Composite; ON, Optic Neuritis.

### Ocular characteristics

**Figure 2:**
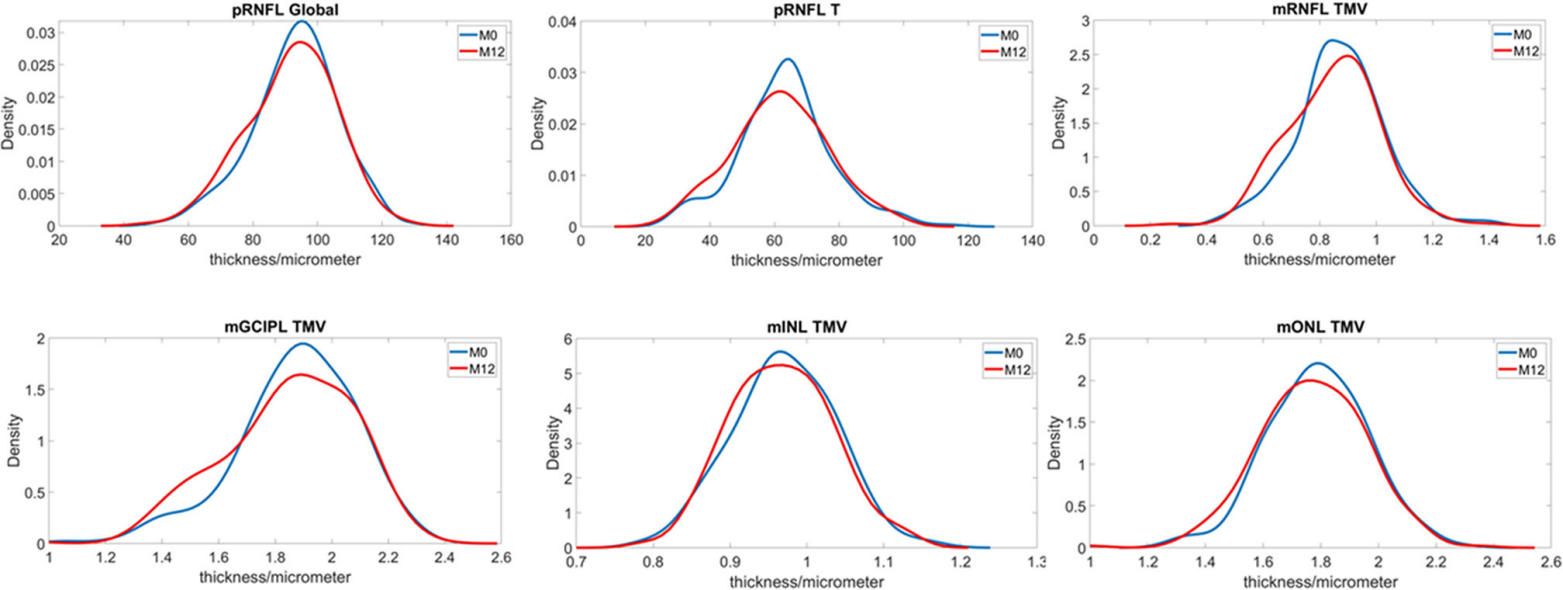
Comparison of retinal thickness between M0 and M12. The line graph shows the changes in the distribution of different retinal layers between M0 and M12. X axis represents retinal measures (micrometre for thickness, cubic micrometre for volume). Y axis is the probability density. mGCIPL, macular ganglion cell inner plexiform complex; mINL, macular inner nuclear layer; mONL, macular outer nuclear layer; mRNFL, macular nerve fibre layer; pRNFL, peripapillary nerve fibre layer. TMV, total macular volume

Of 185 participants included in M0, 142 participants had valid imaging records for protocol 3 with a total of 260 eyes included for the analysis, and 133 had valid imaging records for protocol 4 with a total 241 eyes for the analysis. The measurements of all retinal variables were at similar level between M0 and M12. The measurements and distribution of retinal variables from M0 and M12 were summarised in figure 2 and table 2.

**Table 2:** The comparison of overall retinal parameters between M0 and M12

	M0	M12	P value
pRNFL_Global*	93.087±13.219	91.845±13.801	0.312
pRNFL_Temporal*	63.203±14.421	61.767±14.89	0.281
pRNFL_PMB*	47.83±10.733	46.788±11.162	0.295
mRNFL_TMV†	0.872±0.157	0.848±0.161	0.098
mGCIPL_TMV†	1.874±0.211	1.841±0.243	0.115
mINL_TMV†	0.97±0.067	0.962±0.084	0.229
mONL_TMV†	1.79±0.172	1.765±0.201	0.139

*M0: n=133, 241 eyes; M12: n=137, 245 eyes.

†M0: n=142, 252 eyes; M12: n=139, 260 eyes.

mGCIPL, macular ganglion cell inner plexiform complex; mINL, macular inner nuclear layer; mONL, macular outer nuclear layer; mRNFL, macular nerve fibre layer; PMB, papillomacular bundle; pRNFL, peripapillary nerve fibre layer; TMV, total macular volume.

The proportion of altered profile in pRNFL sectors was summarised in table 3. The highest proportion of BNL was seen in PMB with 13.693% at M0 and 18.776% at M12, and thickness from temporal had the second highest proportion of BNL with 11.203%at M0 and 15.102% at M12. The proportion of BLB was also highest in PMB and temporal with 17.427% and 17.427% at M0 and 17.551% and 19.592% at M12, respectively. The proportion of altered profile was low in all three nasal sectors (NS, N, NI) with less than 2.5% presented with BNL and about 5% presented with BLB, respectively.

**Table 3:** Proportion of altered profile in pRNFL at M0 and M12

	BNL (%)		BLB (%)	
	M0	M12	M0	M12
pRNFL_Global	10.788	14.286	9.129	9.796
pRNFL_PMB	13.693	18.776	17.427	17.551
pRNFL_NS	1.660	2.449	5.394	5.306
pRNFL_N	0.830	0.408	4.979	5.714
pRNFL_NI	0.415	0.000	4.149	3.673
pRNFL_TI	6.639	10.204	6.224	8.980
pRNFL_T	11.203	15.102	17.427	19.592
pRNFL_TS	3.320	6.939	12.448	10.204

BLB, borderline below:a percentile value of <5%; BNL, below normal limit:a percentile value of <1%; N, nasal; NI, nasal inferior; NS, nasal superior; PMB, Papillomacular bundle; pRNFL, peripapillary macular nerve fibre layer; T, temporal; TI, temporal inferior; TS, temporal superior.

## Discussion

MS is associated with significant social and economic costs and as such a better understanding on the pathological processes of the disease is an urgent unmet need. A scalable measure that can accurately predict future disease severity is an important goal. The concept of ‘the eye as a window to the brain’ was raised two decades ago when the RNFL thinning were detected in patients with MS. Over the past 20 years, evidence has emerged indicating an association between inner retinal atrophy and brain atrophy.^14^ A link between retinal parameters and disease activity has also been reported.^6 15 16^ As a low cost, non-invasive technique with few contraindications, OCT has been proposed as a potential tool for disease monitoring and as a complementary measure to MRI parameters. Despite the potential value for the diagnostic process, OCT imaging is not considered as part of the MS diagnostic criteria at the moment due to the inconsistency of the data. A better understanding of the association between the OCT findings and pathological process is needed.

At the whole cohort level, the distribution of retinal measures is generally consistent between M0 and M12. According to a recent prospective longitudinal study on RRMS, in eyes without history of ON, the annual progression rate for pRNFL global thickness is 2.4±2.1 µm for patients with progressive/active disease and 0.5±1.2 µm for stable patients with a cut-off value of 1.5 µm.^17^ Therefore, the retinal measures may be relatively insensitive at a population level over a short study period. Our study further investigated the altered profile in the peripapillary area. It is well established that thinning of pRNFL is associated with MS, and temporal thickness is suggested to be the most sensitive OCT marker in predicting MS.^18^ Our results on the altered profile of pRNFL are in line with previous findings. About 30% of FutureMS participants presented with altered profile in the temporal of pRNFL. In addition, a higher proportion of altered profile was found in PMB indicating the value of PMB for MS. However, when interpreting these data, one should bear in mind that previous attack of ON often result in severe retinal thinning particularly in pRNFL compared with ON-naive eyes. When pooling together, the disparity of retinal measures between the two groups may mask the changes over time. The OCT measurement from FutureMS study have also been analysed longitudinally and cross-sectionally on subcohort based on the history of ON, and will be described in detail elsewhere.

The strengths of the FutureMS OCT imaging dataset are several fold. First of all, FutureMS provides opportunities for deep phenotyping of patients with MS via combining OCT parameters with other biomarkers including MRI imaging, clinical and neurological assessment, laboratory tests as well as genetic data. This enables evaluation of the performance of retinal biomarkers while controlling for other known prognostic factors. Furthermore, FutureMS embraces a comprehensive MRI imaging protocol that captures the microstructure and macrostructure of the posterior visual pathway.^2^ Together with the precise measure of anterior visual pathway obtained from OCT imaging, FutureMS provides an opportunity to elaborate the abnormalities along the afferent visual pathway in MS. Finally, the prospective nature of FutureMS provides opportunities to incorporate new emerging technologies such as OCT angiography in future waves. Combining with the OCT dataset, the extension of the retinal imaging dataset will depict a full picture of the retinal changes in patients with MS, providing a rich dataset for evaluating in vivo visual pathway changes.

Limitations of this study include the exclusion of patients who had rapid DMARD initiation. This may select patients with more benign disease, introducing selection bias which is especially relevant when trying to predict disease course. Furthermore, not all sites were able to recruit patients to the present substudy. However, the demographics of those included here suggest that this population is similar to the overall patient cohort. It is also possible that in this study disease progression is insufficiently advanced to detect via OCT; further work will investigate this prospectively.

We introduce the clinical profile and protocol for the FutureMS study retinal imaging substudy. OCT is a putative non-invasive MS biomarker. The results from this study may facilitate the development of clinical models for the diagnosis and management of MS.

## Data Availability

Data are available on reasonable request. Participants of FutureMS can consent to their deidentified data being shared with any academic or commercial organisations that apply for and receive approval from the independent oversight committee. The FutureMS study welcomes and offers global collaboration. The data are not freely available in the public domain, but proposals and ideas for future collaborations are very welcomed. For more information, the study team (email address: future-ms @ed.ac.uk) with details of the proposed collaboration.

## References

[1] Kearns PKA, Martin SJ, Chang J, et al. FutureMS cohort profile: a Scottish multicentre inception cohort study of relapsing-remitting multiple sclerosis. BMJ Open 2022;12:e058506. 10.1136/bmjopen-2021-058506 PMC9244691 35768080

[2] Meijboom R, Wiseman SJ, York EN, et al. Rationale and design of the brain magnetic resonance imaging protocol for FutureMS: a longitudinal multi-centre study of newly diagnosed patients with relapsing-remitting multiple sclerosis in Scotland. Wellcome Open Res 2022;7:94. 10.12688/wellcomeopenres.17731.1 PMC9971644 36865371

[3] Gabilondo I, Martínez-Lapiscina EH, Martínez-Heras E, et al. Trans-Synaptic axonal degeneration in the visual pathway in multiple sclerosis. Ann Neurol 2014;75:98–107. 10.1002/ana.24030 24114885

[4] Ikuta F, Zimmerman HM. Distribution of plaques in seventy autopsy cases of multiple sclerosis in the United States. Neurology 1976;26:26–8. 10.1212/WNL.26.6_Part_2.26 944889

[5] Balk LJ, Steenwijk MD, Tewarie P, et al. Bidirectional trans-synaptic axonal degeneration in the visual pathway in multiple sclerosis. J Neurol Neurosurg Psychiatry 2015;86:419–24. 10.1136/jnnp-2014-308189 24973342

[6] Knier B, Leppenetier G, Wetzlmair C, et al. Association of retinal architecture, intrathecal immunity, and clinical course in multiple sclerosis. JAMA Neurol 2017;74:847–56. 10.1001/jamaneurol.2017.0377 28460032 PMC5822191

[7] Saidha S, Sotirchos ES, Ibrahim MA, et al. Microcystic macular oedema, thickness of the inner nuclear layer of the retina, and disease characteristics in multiple sclerosis: a retrospective study. Lancet Neurol 2012;11:963–72. 10.1016/S1474-4422(12)70213-2 23041237 PMC3533139

[8] Cellerino M, Cordano C, Boffa G, et al. Relationship between retinal inner nuclear layer, age, and disease activity in progressive MS. Neurol Neuroimmunol Neuroinflamm 2019;6:e596. 10.1212/NXI.0000000000000596 31454778 PMC6705649

[9] Fard MA, Golizadeh A, Yadegari S, et al. Photoreceptor outer nuclear layer thickness changes in optic neuritis follow up. Mult Scler Relat Disord 2019;39:101905. 10.1016/j.msard.2019.101905 31884384

[10] Filgueiras TG, Oyamada MK, Preti RC, et al. Outer retinal dysfunction on multifocal electroretinography may help differentiating multiple sclerosis from neuromyelitis optica spectrum disorder. Front Neurol 2019;10:928. 10.3389/fneur.2019.00928 31507527 PMC6718638

[11] Cruz-Herranz A, Balk LJ, Oberwahrenbrock T, et al. The APOSTEL recommendations for reporting quantitative optical coherence tomography studies. Neurology 2016;86:2303–9. 10.1212/WNL.0000000000002774 27225223 PMC4909557

[12] Tewarie P, Balk L, Costello F, et al. The OSCAR-IB consensus criteria for retinal OCT quality assessment. PLoS One 2012;7:e34823. 10.1371/journal.pone.0034823 22536333 PMC3334941

[13] Grading diabetic retinopathy from stereoscopic color fundus photographs--an extension of the modified Airlie House classification. ETDRS report number 10. Early Treatment Diabetic Retinopathy Study Research Group. Ophthalmology 1991;98:786–806. 2062513

[14] Klistorner A, Sriram P, Vootakuru N, et al. Axonal loss of retinal neurons in multiple sclerosis associated with optic radiation lesions. Neurology 2014;82:2165–72. 10.1212/WNL.0000000000000522 24838786 PMC4113462

[15] Zimmermann HG, Knier B, Oberwahrenbrock T, et al. Association of retinal ganglion cell layer thickness with future disease activity in patients with clinically isolated syndrome. JAMA Neurol 2018;75:1071–9. 10.1001/jamaneurol.2018.1011 29710121 PMC6143115

[16] Chen Q, Jiang H, Delgado S, et al. Longitudinal Study of Retinal Structure, Vascular, and Neuronal Function in Patients With Relapsing-Remitting Multiple Sclerosis: 1-Year Follow-Up. Transl Vis Sci Technol 2021;10:6. 10.1167/tvst.10.6.6 PMC8107487 34111252

[17] Bsteh G, Hegen H, Teuchner B, et al. Peripapillary retinal nerve fibre layer thinning rate as a biomarker discriminating stable and progressing relapsing-remitting multiple sclerosis. Eur J Neurol 2019;26:865–71. 10.1111/ene.13897 30614590

[18] Birkeldh U, Manouchehrinia A, Hietala MA, et al. The temporal retinal nerve fiber layer thickness is the most important optical coherence tomography estimate in multiple sclerosis. Front Neurol 2017;8:675. 10.3389/fneur.2017.00675 29326643 PMC5733353

