# Supplementary material for: Longitudinal retinal imaging study of newly diagnosed relapsing-remitting multiple sclerosis in Scottish population: baseline and 12 months follow-up profile of FutureMS retinal imaging cohort
Source: BMJ Open Ophthalmol. 2022 Jul 22;7(1):e001024. doi: 10.1136/bmjophth-2022-001024 (PMC9315911; doi:10.1136/bmjophth-2022-001024)
Supplement: Supplementary data [file bmjophth-2022-001024supp001.pdf]

Supplement materials

Supplementary Table 1 OCT device information participating in FutureMS retinal imaging study

	Edinburgh		Glasgow
	before 2018.8.28	after 2018.8.28	
Camera model	Spectralis OCT	Spectralis OCT	Spectralis OCT
Camera model code	S2610-/S2001A	S2610-/S2001A	S2400-B
HRA camera FW version	2.4.3.0	2.6.3.0	2.6.3.0
Acquisition Software version	6.0.11.0	6.9.4.0	6.9.4.0

Supplementary Table 2 FutureMS retinal imaging study scanning protocols

	Protocol 1<br>H line scan	Protocol 2<br>V line scan	Protocol 3<br>P pole volume scan	Protocol 4<br>RNFL circle scan
Image acquisition module	Retina	Retina	Retina	N-site
Scan pattern	Single line	Single line	Volume	Circle
Scan orientation	Horizontal	Vertical	7° horizontal	
Scan length	30°	30°	30° × 25°	12°
Scan centre	fovea	fovea	fovea	Optic nerve head
Scan preset	ART [100]	ART [100]	ART [12]<br>Posterior pole<br>Number of Sections [61]<br>Section spacing [120µm]	ART [100]

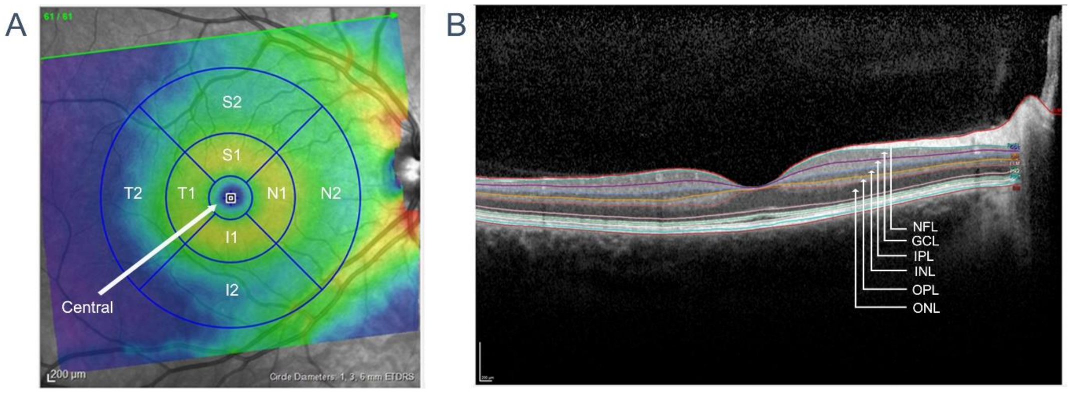

Supplementary Figure 1 The structure of macular area obtained via FutureMS OCT imaging Protocol 3

A. An example of thickness analysis from posterior pole volume scan based on ETDRS grid. False color tomographic image represent the scanned area reconstructed from 61 line scan. The nine-sector ETDRS chart centred on fovea shows area where the measurements were obtained. The three circles from innermost to outermost represent the areas within 1mm, 3mm and 6mm of fovea respectively. Central: average thickness of the area within 1mm. T1, S1, N1,and I1 represent the average thickness of temporal, superior, nasal, inferior area of the area within 3mm. T2, S2, N2, and I2 indicate the corresponding area within 6mm. TMV represents the total volume of the 6mm zone (the area within the outermost ring). B:The cross section view of the line cross the fovea and the segmentation of retinal layers. NFL: nerve fibre layer; GCL: ganglion cell layer; IPL: inner plexiform layer; INL: inner nuclear layer; OPL: outer plexiform layer; ONL: outer nuclear layer. Scale bar: 200µm.

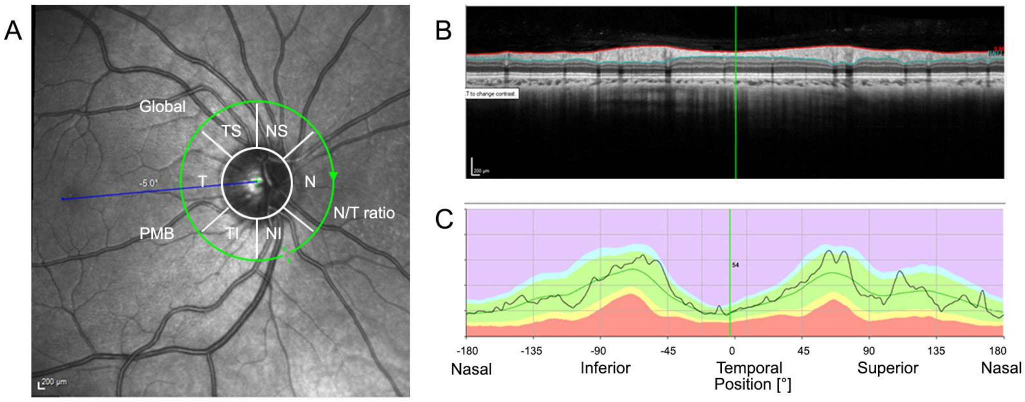

Supplementary Figure 2 The structure of pRNFL circle scan by FutureMS OCT imaging Protocol 4

A. An example of the pRNFL circle scan centred on optic disc and the illustration on classification results including global average thickness of the circle scan, the standard six sectors, papillomacular bundle (PMB) as well as N/T ratio. The white circle demonstrates the edge of the optic disc and the green circle depicts the position of the scan. T: Temporal; TS: Temporal superior; NS: Nasal superior; N: Nasal; NI: Nasal inferior; TI: temporal inferior; B. The cross section view of pRNFL with contour line. The red line indicates the internal limiting membrane (ILM), the upper boundary of NFL and the green line indicates the bottom of the nerve fibre layer. C. The black line graph demonstrating the profile of pRNFL measured along the circle scan against normal range. The green area indicates the 5th and 95th percentile of normal range. The light blue area represents the range above 95th percentile of normal distribution. The yellow band and red band represent the range between 1st and 5th percentile and the range below 1st percentile respectively. Scale bar: 200µm.
